# Relationship of prefrontal cortex activity with anhedonia and cognitive function in major depressive disorder: an fNIRS study

**DOI:** 10.3389/fpsyt.2024.1428425

**Published:** 2024-09-19

**Authors:** Huanhuan Fan, Qing Li, Yue Du, Yushun Yan, Rongjun Ni, Jinxue Wei, Liansheng Zhao, Xiao Yang, Xiaohong Ma

**Affiliations:** ^1^ Mental Health Center and Institute of Psychiatry, West China Hospital, Sichuan University, Chengdu, China; ^2^ Mental Health Center, West China Hospital, Sichuan University, Chengdu, Sichuan, China

**Keywords:** anhedonia, hedonic processing, major depressive disorder, functional near-infrared spectroscopy, sustained attention, working memory

## Abstract

**Background:**

Major depressive disorder (MDD) is associated with deficits in cognitive function, thought to be related to underlying decreased hedonic experiences. Further research is needed to fully elucidate the role of functional brain activity in this relationship. In this study, we investigated the neurofunctional correlate of the interplay between cognitive function and hedonic experiences in medication-free MDD using functional near-infrared spectroscopy (fNIRS).

**Methods:**

We examine differences of brain activation corresponding to the verbal fluency test (VFT) between MDD patients and healthy controls (HCs). Fifty-six MDD patients and 35 HCs underwent fMRI scanning while performing the VFT. In exploratory analyses, cognitive performance, as assessed by the Cambridge Neuropsychological Test Automated Battery (CANTAB), four dimensions of hedonic processing (desire, motivation, effort, and consummatory pleasure) measured by the Dimensional Anhedonia Rating Scale (DARS), and relative changes in oxygenated hemoglobin concentration during the VFT were compared across groups.

**Results:**

Patients with MDD demonstrated impairments in sustained attention and working memory, accompanied by lower total and subscale scores on the DARS. Compared to healthy controls, MDD patients exhibited reduced activation in the prefrontal cortex (PFC) during the VFT task (t = 2.32 to 4.77, *p* < 0.001 to 0.02, FDR corrected). DARS motivation, desire, and total scores as well as sustained attention, were positively correlated with activation in the dorsolateral PFC and Broca’s area (*p* < 0.05, FDR corrected).

**Conclusions:**

These findings indicate that changes in prefrontal lobe oxygenated hemoglobin levels, a region implicated in hedonic motivation and cognitive function, may serve as potential biomarkers for interventions targeting individuals with MDD. Our results corroborate the clinical consensus that the prefrontal cortex is a primary target for non-invasive neuromodulatory treatments for depression.

## 1 Introduction

Major Depressive Disorder (MDD) is a chronic and severe mental illness that significantly impairs an individual’s life. Core symptoms encompass a persistent low mood (increased negative emotions) and profound anhedonia (decreased positive emotions), a state where once-pleasurable activities no longer evoke pleasure or motivation. This clinical presentation of MDD is often accompanied by a range of cognitive deficits, such as difficulties in executive control, attention, and working memory, which can significantly hinder an individual’s daily functioning and overall productivity (1, 2). Numerous studies have consistently demonstrated that a substantial proportion of depressed patients suffer from varying degrees of anhedonia, with a significant range spanning from approximately 37% to 75% of MDD patients reporting significant levels of anhedonia (3–6). Anhedonia not only predicts a blunted response to serotonin-modulating antidepressants and psychological treatments but is also associated with a higher risk of future suicidal behaviors and poorer functional outcomes (7–10). When individuals undergoing depressive episodes concurrently experience anhedonia, their clinical symptoms tend to be more severe (11). Limited evidence suggest that behavioral activation (BA) and cognitive-behavioral therapy (CBT) may be partially effective in alleviating anhedonia during acute period, but there is no further improvement during subsequent visits (12). Therefore, more research is essential to understand the neural correlates of anhedonia and identify biomarkers that have important implications for new treatment targets and predictors of treatment response.

The prefrontal cortex (PFC) is central to reward processing, with distinct subregions contributing to different aspects of reward-related behavior. The ventromedial PFC (vmPFC) is responsible for assigning value to rewards, while the lateral PFC modulates these valuations to facilitate self-control and future-oriented decisions (13, 14). Hypoactivation of PFC, commonly observed in MDD, impairs the detection of stimulus significance and disrupts reward processing (15–17). Specifically, reduced medial orbitofrontal cortex (mOFC) activation in response to rewards correlates with anhedonia and dysfunction of the dorsolateral PFC (dlPFC) contribute to reward-seeking deficits in MDD (16, 17). Noninvasive brain stimulation techniques hold promise for ameliorating these reward impairments (17–20).

Previous studies have investigated the relationship between anhedonia and cognitive deficits. In a relatively large sample, McIntyre and his team found a strong association between anhedonia and subjective cognitive symptoms, even after controlling for illness severity (21). Similarly, other studies suggest that enhanced cognitive control may help individuals manage negative information, reducing rumination and increasing attention to positive information (22, 23). Paradoxically, a comprehensive analysis of outpatients suffering from MDD revealed no association between reward learning and anhedonia (24). The investigators postulated that this disparity might stem from the anhedonia is a complex concept, encompassing different aspects of pleasure and reward processing (25). Given the significance of anhedonia in MDD and its relationship with cognitive deficits, it is critical to identify the specific PFC regions that are specifically associated with this symptom in affected individuals.

Functional near-infrared spectroscopy (fNIRS) offers a relatively non-invasive, well-tolerated, convenient, and cost-effective alternative method. fNIRS is an optical method used to monitor the fluctuations in the levels of oxygenated (oxy-) and deoxygenated hemoglobin (deoxy-Hb), similar to blood oxygenation level dependent (BOLD) signal is measured in fMRI research (26, 27). The reliability and feasibility of fNIRS in characterizing brain activation and functional connectivity have been demonstrated by multiple studies (28, 29). The Verbal Fluency Task (VFT) is a representative cognitive task in fNIRS research which requires participants to generate a maximum number of non-repetitive words based on a given phonemic cue (30). It demands a wide variety of functions from the bilateral frontotemporal regions including memory retrieval, attentional control, executive function, and information processing speed (31). Previous fNIRS studies have detected the hemodynamic changes in these regions during the VFT, which are believed to be linked to the sleep quality and cognitive deficits observed in MDD patients (32, 33). Therefore, the VFT is widely employed to elucidate brain dysfunctions associated with psychiatric disorders, as it effectively highlights deficits in critical cognitive functions (34).

This study aimed to comprehensively characterize hedonic processing in MDD patients and examine its association with alterations in PFC activity and cognitive function. Building upon previous studies linking altered PFC activation to cognitive impairments and aberrant reward responses in MDD (15–17, 35, 36), we hypothesized that PFC activity plays a central role in these relationships.

## 2 Methods

### 2.1 Participants

Depressed subjects were recruited from the Department of Psychiatry in West China Hospital, Sichuan University, while comparison subjects were recruited from the community and media advertising. MDD participants who aged between 16 and 55 years old, had a DSM-V diagnosis of MDD, a score ≥13 on the Beck Depression Inventor (BDI-21) and a score ≥14 on the 17-item Hamilton Depression Rating Scale (HAMD-17) (37–39). Exclusion criteria included psychotropic medication in the last 2 weeks (fluoxetine: 6 weeks), current or past history of MDD with psychotic features, and presence of other Axis I diagnosis (including lifetime substance dependence and substance use disorders in the last year), with the exception of anxiety disorders. Healthy controls (HCs) reported no medical or neurological illness, no current or past psychopathology, and no psychotropic medications. All subjects were right-handed.

### 2.2 Clinical and cognitive assessments

We assessed cognitive function using the Cambridge Neuropsychological Testing Automated Battery (CANTAB) (http://www.cambridgecognition.com/cantab/ neurocognitive-tests). It is noteworthy that our cognitive battery included only Rapid Visual Information Processing (RVP) and Spatial Working memory (SWM) to test sustained attention and working memory, two key components of cognitive control, as our primary interest lies in studying cognitive functions in depression. Previous literature also used these indicators to represent the above cognitive functions (40, 41). Participants completed the Chinese version of Dimensional Anhedonia Rating Scale (DARS), a 17-item self-report instrument measures four subs-cales based on component of reward processing including hedonic desire, motivation, effort and consume symptoms, with higher scores indicating more motivation and pleasure across the week (42–44).

### 2.3 Verbal fluency task

Participants completed one trial of a Chinese-language version of the letter VFT, which is considered a reliably neuropsychological test to measure prefrontal abnormalities in major psychiatric disorders (28). The subjects were asked to generate as many words as possible. The words generated were marked as either correct or incorrect responses, and the number of correct words represented the subject’s performance score in the task. Each trial consisted of a 45-s pre-task baseline period, a 60-s task period subdivided into three 20-s blocks and finally, a 72-s post-task baseline period.

### 2.4 Instrument and analysis of fNIRS signals

**Figure 1:**
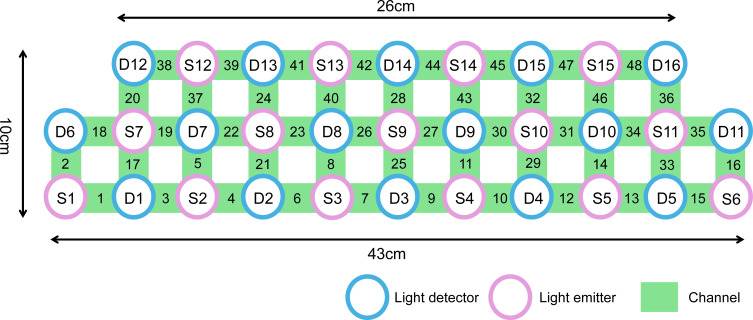
Channel distribution of the functional near-infrared spectroscopy (fNIRS) measurement.

We used a NIRS instrument (Danyang Huichuang) with 15 light sources (wavelengths: 730nm, 808nm and 850 nm) and 16 detectors. The locations of the sources and detectors were digitized using a Polhemus Fast Trak 3D digitizer. The area measured between the pair of probes was defined as a channel. It generated 48 measurement channels with a fixed source-detector distance of 3 cm covering the frontal and temporal lobes. Our channel distribution diagram of fNIRS was shown in **Figure 1**. The probes were placed on the head along the Fp1-Fp2 line according to the relevant standard positions of the international 10-20 system. The middle inferior optode was placed over Fpz, and the inferior row of optodes was oriented towards T3 and T4, respectively. Near-infrared light of three different wavelengths was used to detect the concentration signals of oxy-Hb and deoxy-Hb at a sampling rate of 11 Hz. In this experiment, we defined the PFC area consisting of 28 channels (Channel4/6/7/8/9/10/11/12/21/22/23/24/25/26/27/28/29/30/32/37/39/40/41/43/44/45/46/47, covering Brodmann (BA) 9/10/11/45/46/47, which is as a region of interest (ROI). The raw fNIRS signals were preprocessed with functions of the Homer2 processing package based on Matlab 2013b software. The intensity data were converted into optical density changes and transformed to relative changes of oxy-Hb/deoxy-Hb by taking the logarithm of the signal (function: hmrR_Itensity2OD). These changes are transformed into concentration changes of oxy-Hb and deoxy-Hb as indicators for brain activity by means of a modified Beer–Lambert Law. Oxy-Hb signals from 5 s before to 60 s after the VFT task in each trial were retained for subsequent analysis. Through the toolkit of NIRS-SPM, the general linear regression model (GLM) analysis with hemodynamic response curve to model the oxy-Hb response during VFT condition was performed to produce beta values as the individual VFT-related activity. We did this in the same as a previous study (45). The beta values of all channel were z-transformed prior to analysis in order to normalize their distributions.

### 2.5 Statistical analyses

IBM Statistics 26.0 software was used for statistical analyses, the significance was defined as *P* < 0.05. Two sample t-tests were performed to test for group differences in sociodemographic, clinical variables and cognitive function, while chi-square test was performed for categorical variable. *P*-values obtained from the statistical tests were corrected using false discovery rate (FDR) method to correct multiple comparisons. Pearson’s correlation was conducted to assess the potential relationships between cognitive function and DARS total scores, four subscale scores including hedonic consume, desire, motivation, effort and beta values in the channels that showed significant between-group differences.

## 3 Results

### 3.1 Demographic and clinical characteristics

The final sample included 56 MDD and 35 demographically matched comparison subjects (**Table 1**). MDD subjects were moderately depressed, as assessed by Beck Depression Inventory (BDI-21) (31.9 ± 12.3) and 17-item HAMD (21.3 ± 4.3) scores. Among the MDD subjects, 35 (86%) had never received antidepressants and 21 (14%) reported prior antidepressant use. There were no significant differences in age and gender between the MDD groups and HCs. The MDD patients showed poorer VFT performance than the HCs, and the mean number of the correct items there was significantly lower.

**Table 1:** Demographic, clinical characteristics and Task Performance of the sample.

Variables	MDD (N = 56)	HCs (N = 35)	Statistic	*P* value
**Age (years)**	27.0 ± 9.0	28.2 ± 8.7	0.64	0.53^b^
**Gender (M/F)**	17/39	15/20	1.48	0.22^a^
**Total HAMD score**	21.3 ± 4.3	–	–	–
**Total BDI score**	31.9 ± 12.3	3.9 ± 4.4	-12.85	<0.001^b^
**Age of illness onset**	23.5 ± 8.9	–	–	–
**Total DARS score**	30.4 ± 16.5	58.2 ± 10.5	8.88	<0.001^b^
**DARS_consume** **DARS_effort** **DARS_desire** **DARS_motivation** **VFT score**	8.0 ± 4.2 6.6 ± 4.2 11.7 ± 6.0 4.1 ± 3.2 10.5 ± 3.9	14.1 ± 2.3 13.2 ± 2.7 21.1 ± 3.5 9.7 ± 2.6 13.2 ± 4.6	7.88 8.22 8.49 8.81 3.08	<0.001^b^ <0.001^b^ <0.001^b^ <0.001^b^ 0.003^b^

Data presented as mean (SD) for continuous variables and N for categorical variables. M, male; F, female. BDI-II, Beck Depression Inventory-II; DARS, Dimensional Anhedonia Rating Scale; CON, hedonic consume score of DARS; EFF, hedonic effort score of DARS; DES, hedonic desire score of DARS; MOT, hedonic motivation score of DARS; VFT, verbal fluency task.

^a^The P value was obtained by chi-square test.

^b^The P values were obtained by two-sample t-test.

### 3.2 Group comparisons of cognitive function, anhedonia and PFC activation during the VFT task

**Figure 2:**
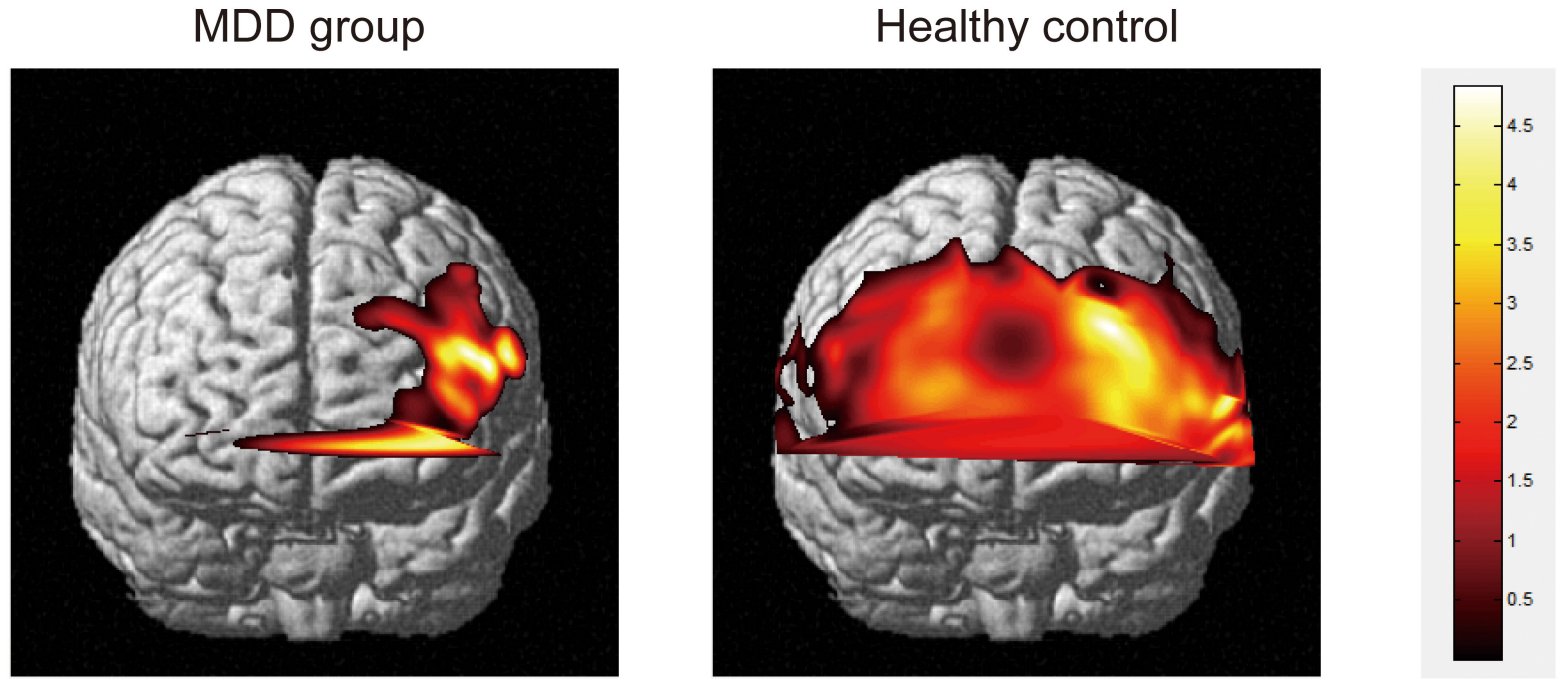
Activation plots of brain regions corresponding to mean oxygen-hemoglobin β values across all channels during the VFT task (*p* < 0.05) in frontal view. Right vertical bar from top to bottom with color change represents decreasing activation.

MDD patients exhibited worse sustained attention, working memory, and significantly higher levels of anhedonia compared to HCs (**Tables 1**, **2**). MDD patients showed significantly decreased activity in the nineteen channels of the PFC (CH4, CH6-10, CH21-30, CH32, CH40 and CH41 in the ventral lateral PFC and part of the dlPFC; *t* = 2.32-4.77, FDR *p <*0.001- FDR *p* = 0.02) during the VTF task when compared with HCs (**Figure 2**).

**Table 2:** The results of sustained attention and working memory comparisons.

Variables	MDD (N = 56)	HCs (N = 35)	t	*P* value
**RVP_A’**	0.9 ± 0.1	0.9 ± 0.0	-4.14	<0.001
**RVP**_**TH**	16.1 ± 5.3	19.9 ± 5.3	-3.32	0.001
**RVP**_**TM**	10.9 ± 5.3	7.1 ± 5.3	3.30	0.001
**RVP**_**PH**	0.60 ± 0.2	0.7 ± 0.2	-3.31	0.001
**SWM**_**BE**	30.1 ± 19.5	14.7 ± 12.9	4.13	<0.001
**SWM**_**TE**	31.0 ± 19.6	15.9 ± 13.9	3.97	<0.001
**SWM**_**strategy**	33.6 ± 5.5	31.5 ± 4.9	-1.81	0.07

Data presented as mean (SD) for continuous variables. RVP, Rapid Visual Information Processing; A’, is the signal detection measure of sensitivity to the target, regardless of response tendency (range 0.00 to 1.00; bad to good); TH, total hits; TM, total misses; PH, probability of hit; SWM, spatial working memory; BE, times the subject revisits a box in which a token has previously been found; TE, This is the number of times a box is selected that is certain not to contain a blue token and therefore should not have been visited by the subject; strategy: higher scores indicating inferior neurocognitive performance.

### 3.3 Correlations among anhedonia, prefrontal activity and cognitive performance in the patients group

**Figure 3:**
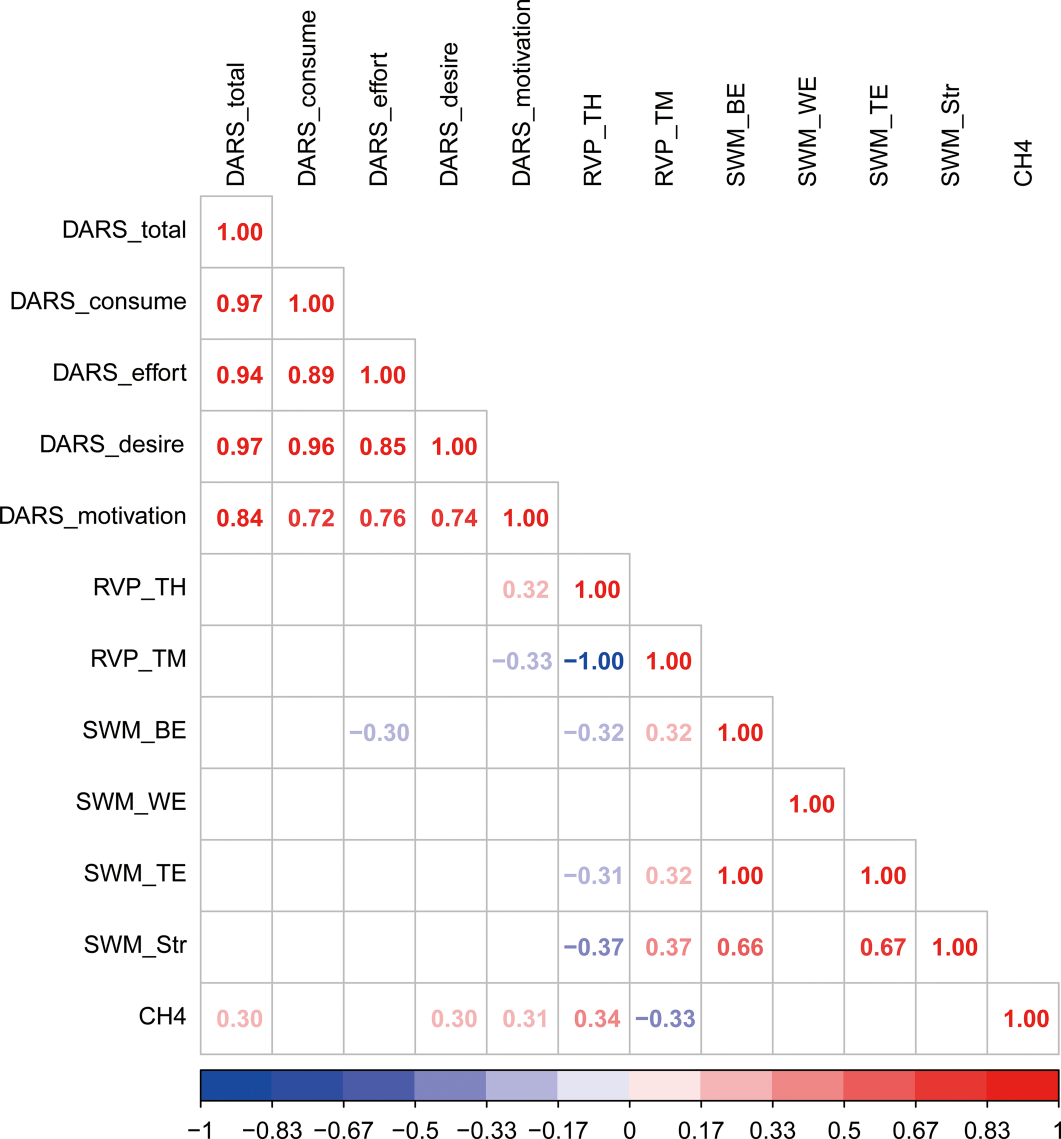
Correlations among anhedonia, prefrontal activity and cognitive performance in the patients group.

Patients with MDD who reported lower motivation scores on the DARS demonstrated poorer performance on the RVP test (FDR-corrected p < 0.05). CH4 beta values were positively correlated with DARS motivation, desire, and total scores (FDR-corrected p < 0.05), as well as with RVP test performance (FDR-corrected p < 0.05). These findings are detailed in **Figure 3** and **Supplementary Tables 1-3**.

## 4 Discussion

Our primary finding is that medication-free current MDD is characterized by reduced hedonic experiences, poorer performance on tests of sustained attention and working memory, as well as decreased prefrontal activity during the VFT task. Notably, our findings highlight significant anhedonia in MDD patients, characterized by diminished hedonic desire, motivation, effort and consume suggesting multifaceted impairments in reward processing in depression. Furthermore, our results indicate significant deficits in sustained attention, working memory and verbal fluency, evidenced by lower accuracy, increased error rates, reduced sensitivity to targets and diminished word production within a specified time frame. In summary, our study highlights the complex interplay between cognitive deficits, anhedonia, and neural dysfunction in MDD, contributing to a deeper understanding of the multifaceted nature of depressive disorders.

In line with our findings, a large body of studies indicates that MDD patients exhibit cognitive impairments in domains such as processing speed, attention, memory, verbal learning, as well as executive function (46, 47). Cognitive function is particularly relevant to educational and occupational pursuits, with a greater impact on workplace performance than the severity of depression symptoms (48, 49). While correlations between estimated beta values and anhedonia scores suggest the potential of the VFT task to reflect brain dysfunction in MDD, our study did not find significant associations between VFT performance, anhedonia, or brain activity within our sample.

In our study, MDD participants exhibiting decreased hedonic experiences including decreased desire, motivation, and overall pleasure, showed reduced PFC activity primarily within the CH4, comprising the dlPFC and Broca’s area (corresponding to BA46 and 45). Patients experiencing the most severe anhedonia are expected to exhibit more pronounced dysfunction in reward systems (50–53). It is important to note the nucleus accumbens and orbitofrontal cortex (OFC) are crucial for pleasure perception, with both regions exhibiting reduced activity in MDD individuals (54–58). The OFC transmits reward value information to the anterior cingulate cortex (ACC), which calculates the effort needed to attain rewards (59, 60). The anterior vmPFC and dlPFC then process this information for complex decision-making (60–64). Our findings were partially consistent with previous research, which reported decreased dlPFC activation and increased striatal activity in depressed subjects during learning tasks (65). Conversely, animal research indicates heightened dlPFC activity in response to motivating goals (66). In parallel with the notion of prefrontal-basal ganglia dysfunction in reward processing, our observed decreased dlPFC activity suggests potential underlying deficits in these interconnected systems (57). However, this study did not explicitly examine these relationships. Our findings extend previous research by highlighting the involvement of the dlPFC in reward processing and its potential as a therapeutic target. Additionally, we observed altered activation in Broca’s area, located within the right inferior frontal gyrus (rIFG), a region implicated in inhibitory control (67). As a component of the executive control network, the rIFG also modulates the reward system (68). Our subjects are supposed to activate this region when performing the VFT task to generate more words while avoid repetition. Notably, this region has been previously linked to altered responses to facial expressions in individuals with major depressive disorder (69, 70). Given the limited existing evidence, further research is necessary to elucidate the role of the rIFG in mental disorders.

Our findings indicate a significant correlation between deficits in the motivational aspects of hedonic processing and impaired sustained attention in MDD. This suggests that individuals with depression exhibit a decreased drive towards goal-oriented behavior, coupled with an overemphasized tendency to avoid, thereby hindering their ability to flexibly adapt attention to emotional stimuli (71–73). In line with the perspective that depression is characterized by fundamental motivational deficits, patients with MDD exert less cognitive effort for reward compared to controls (74–76).

Extensive research, including fNIRS studies, has demonstrated the PFC’s critical role in a wide range of cognitive functions and its vulnerability to cognitive impairment (77–79). Lateral PFC regions mediate selective attention by facilitating the processing of goal-relevant information and inhibiting the processing of irrelevant information (80, 81). This study found correlation between reduced activity in PFC and impaired sustained attention in patients with MDD. This is also in keeping with another finding that showed people with depression were unable to recruit frontal areas during cognitive control while exhibiting heightened activation during an emotional task conducted separately (82). The observed reduced activation in the right dlPFC suggests that a cognitive remediation training, which boosts attention-related neural activity in the PFC, has the potential to improve cognition among individuals with MDD (83).

While specialized measures such as the Temporal Experience of Pleasure Scale and the Snaith-Hamilton Pleasure Scale exist for assessing anhedonia, these tools primarily focus on predefined hedonic experiences (4, 84). Given the complex nature of reward processing involved in anhedonia, a more comprehensive approach is necessary. This study utilized a comprehensive instrument designed to capture a wide range of hedonic processes related to personal behaviors and experiences, providing a more nuanced understanding of the subjective experience of pleasure.

Several limitations of this study should be considered. First, the sample size was relatively small, necessitating replication with a larger cohort to confirm findings. Second, participants underwent a two-hour clinical assessment before research tasks, raising the possibility of fatigue effects. Future studies employing a variety of cognitive tasks are warranted to further elucidate the relationship between cognitive, anhedonia and brain functional variables. Additionally, while participants were medication-free for at least two weeks, the potential long-term effects of antidepressants or antipsychotics on neuronal function and cognitive performance cannot be entirely ruled out.

## 5 Conclusions

These findings enhance our comprehension of the underlying pathophysiology of anhedonia and its interplay with cognition in MDD, as well as its association with brain activity. Our study provides further evidence supporting the notion that investigating the relationship between hedonic impairment and neural correlates of cognitive deficits in MDD may elucidate the clinical consensus identifying the prefrontal cortex as a primary target for non-invasive neuromodulation in depression.

## Data Availability

The raw data supporting the conclusions of this article will be made available by the authors, without undue reservation.

## References

[1] Roiser JP Elliott R Sahakian BJ. Cognitive mechanisms of treatment in depression. Neuropsychopharmacology. (2012) 37:117–36. doi: 10.1038/npp.2011.183 PMC3238070 21976044

[2] Culpepper L Muskin PR Stahl SM. Major depressive disorder: understanding the significance of residual symptoms and balancing efficacy with tolerability. Am J Med. (2015) 128:S1–S15. doi: 10.1016/j.amjmed.2015.07.001 26337210

[3] Cao B Park C Subramaniapillai M Lee Y Iacobucci M Mansur RB. The efficacy of vortioxetine on anhedonia in patients with major depressive disorder. Front Psychiatry. (2019) 10:17. doi: 10.3389/fpsyt.2019.00017 30766492 PMC6365446

[4] Franken IH Rassin E Muris P. The assessment of anhedonia in clinical and non-clinical populations: further validation of the Snaith-Hamilton Pleasure Scale (SHAPS). J Affect Disord. (2007) 99:83–9. doi: 10.1016/j.jad.2006.08.020 16996138

[5] Tang W Liu H Chen L Zhao K Zhang Y Zheng K. Inflammatory cytokines, complement factor H and anhedonia in drug-naïve major depressive disorder. Brain Behav Immun. (2021) 95:238–44. doi: 10.1016/j.bbi.2021.03.022 33794316

[6] Pelizza L Ferrari A. Anhedonia in schizophrenia and major depression: state or trait? Ann Gen Psychiatry. (2009) 8:22. doi: 10.1186/1744-859X-8-22 19811665 PMC2764701

[7] Kang L Wang W Zhang N Yao L Tu N Feng H. Anhedonia and dysregulation of an angular gyrus-centred and dynamic functional network in adolescent-onset depression. J Affect Disord. (2023) 324:82–91. doi: 10.1016/j.jad.2022.12.057 36581179

[8] Dunlop K Rizvi S Hassel S Strother SC Liston C. Pre-treatment resting-state functional connectivity related to anhedonia and anxiety are associated with antidepressant response to escitalopram and adjunct aripiprazole. Biol Psychiatry. (2020) 87:S431. doi: 10.1016/j.biopsych.2020.02.1098

[9] Sandman CF Craske MG. Psychological treatments for anhedonia. Curr Top Behav Neurosci. (2022) 58:491–513. doi: 10.1007/7854_2021_291 34935116

[10] Wong S Le GH Phan L Rhee TG Ho R Meshkat S. Effects of anhedonia on health-related quality of life and functional outcomes in major depressive disorder: A systematic review and meta-analysis. J Affect Disord. (2024) 356:684–98. doi: 10.1016/j.jad.2024.04.086 38657767

[11] Ducasse D Loas G Dassa D Gramaglia C Zeppegno P Guillaume S. Anhedonia is associated with suicidal ideation independently of depression: A meta-analysis. Depress Anxiety. (2018) 35:382–92. doi: 10.1002/da.2018.35.issue-5 29232491

[12] Alsayednasser B Widnall E O’Mahen H Wright K Warren F Ladwa A. How well do Cognitive Behavioural Therapy and Behavioural Activation for depression repair anhedonia? A secondary analysis of the COBRA randomized controlled trial. Behav Res Ther. (2022) 159:104185. doi: 10.1016/j.brat.2022.104185 36371903

[13] Pelletier G Fellows LK. A critical role for human ventromedial frontal lobe in value comparison of complex objects based on attribute configuration. J Neurosci. (2019) 39:4124–32. doi: 10.1523/JNEUROSCI.2969-18.2019 PMC6529858 30867258

[14] Ballard IC Aydogan G Kim B McClure SM. Causal evidence for the dependence of the magnitude effect on dorsolateral prefrontal cortex. Sci Rep. (2018) 8:16545. doi: 10.1038/s41598-018-34900-y 30410093 PMC6224465

[15] Smoski MJ Felder J Bizzell J Green SR Ernst M Lynch TR. fMRI of alterations in reward selection, anticipation, and feedback in major depressive disorder. J Affect Disord. (2009) 118:69–78. doi: 10.1016/j.jad.2009.01.034 19261334 PMC2745481

[16] Xie C Jia T Rolls ET Robbins TW Sahakian BJ Zhang J. Reward versus nonreward sensitivity of the medial versus lateral orbitofrontal cortex relates to the severity of depressive symptoms. Biol Psychiatry Cognit Neurosci Neuroimaging. (2021) 6:259–69. doi: 10.1016/j.bpsc.2020.08.017 33221327

[17] Bi R Zhao Y Li S Xu F Peng W Tan S. Brain stimulation over the left DLPFC enhances motivation for effortful rewards in patients with major depressive disorder. J Affect Disord. (2024) 356:414–23. doi: 10.1016/j.jad.2024.04.064 38640975

[18] Downar J Geraci J Salomons TV Dunlop K Wheeler S McAndrews MP. Anhedonia and reward-circuit connectivity distinguish nonresponders from responders to dorsomedial prefrontal repetitive transcranial magnetic stimulation in major depression. Biol Psychiatry. (2014) 76:176–85. doi: 10.1016/j.biopsych.2013.10.026 24388670

[19] Duprat R De Raedt R Wu GR Baeken C. Intermittent theta burst stimulation increases reward responsiveness in individuals with higher hedonic capacity. Front Hum Neurosci. (2016) 10:294. doi: 10.3389/fnhum.2016.00294 27378888 PMC4910023

[20] Duprat R Wu GR De Raedt R Baeken C. Accelerated iTBS treatment in depressed patients differentially modulates reward system activity based on anhedonia. World J Biol Psychiatry. (2018) 19:497–508. doi: 10.1080/15622975.2017.1355472 28789578

[21] McIntyre RS Woldeyohannes HO Soczynska JK Maruschak NA Wium-Andersen IK Vinberg M. Anhedonia and cognitive function in adults with MDD: results from the International Mood Disorders Collaborative Project. CNS Spectr. (2016) 21:362–6. doi: 10.1017/S1092852915000747 26714651

[22] Hsu KJ Beard C Rifkin L Dillon DG Pizzagalli DA Björgvinsson T. Transdiagnostic mechanisms in depression and anxiety: The role of rumination and attentional control. J Affect Disord. (2015) 188:22–7. doi: 10.1016/j.jad.2015.08.008 PMC4765997 26340079

[23] Vasey MW Harbaugh CN Mikolich M Firestone A Bijttebier P. Positive affectivity and attentional control moderate the link between negative affectivity and depressed mood. Pers Individ Dif. (2013) 54:802–7. doi: 10.1016/j.paid.2012.12.012

[24] Liao A Walker R Carmody TJ Cooper C Shaw MA Grannemann BD. Anxiety and anhedonia in depression: Associations with neuroticism and cognitive control. J Affect Disord. (2019) 245:1070–8. doi: 10.1016/j.jad.2018.11.072 PMC9667857 30699849

[25] Rizvi SJ Pizzagalli DA Sproule BA Kennedy SH. Assessing anhedonia in depression: Potentials and pitfalls. Neurosci Biobehav Rev. (2016) 65:21–35. doi: 10.1016/j.neubiorev.2016.03.004 26959336 PMC4856554

[26] Leff DR Orihuela-Espina F Elwell CE Athanasiou T Delpy DT Darzi AW. Assessment of the cerebral cortex during motor task behaviours in adults: a systematic review of functional near infrared spectroscopy (fNIRS) studies. NeuroImage. (2011) 54:2922–36. doi: 10.1016/j.neuroimage.2010.10.058 21029781

[27] Gramigna V Pellegrino G Cerasa A Cutini S Vasta R Olivadese G. Near-infrared spectroscopy in gait disorders: is it time to begin? Neurorehabilitation Neural Repair. (2017) 31:402–12. doi: 10.1177/1545968317693304 28196453

[28] Wei Y Chen Q Curtin A Tu L Tang X Tang Y. Functional near-infrared spectroscopy (fNIRS) as a tool to assist the diagnosis of major psychiatric disorders in a Chinese population. Eur Arch Psychiatry Clin Neurosci. (2021) 271:745–57. doi: 10.1007/s00406-020-01125-y 32279143

[29] Ferrari M Quaresima V. A brief review on the history of human functional near-infrared spectroscopy (fNIRS) development and fields of application. NeuroImage. (2012) 63:921–35. doi: 10.1016/j.neuroimage.2012.03.049 22510258

[30] Kumar V Shivakumar V Chhabra H Bose A Venkatasubramanian G Gangadhar BN. Functional near infra-red spectroscopy (fNIRS) in schizophrenia: A review. Asian J Psychiatr. (2017) 27:18–31. doi: 10.1016/j.ajp.2017.02.009 28558892

[31] Henry JD Crawford JR. A meta-analytic review of verbal fluency performance following focal cortical lesions. Neuropsychology. (2004) 18:284–95. doi: 10.1037/0894-4105.18.2.284 15099151

[32] Li Y Li X Zhaung W Yu C Wei S Li Y. Relationship between cognitive function and brain activation in major depressive disorder patients with and without insomnia: A functional near-infrared spectroscopy (fNIRS) study. J Psychiatr Res. (2024) 169:134–41. doi: 10.1016/j.jpsychires.2023.11.002 38039687

[33] Xu H Wang Y Wang YM Cao Y Li P Hu Y. Insomniacs show greater prefrontal activation during verbal fluency task compared to non-insomniacs: a functional near-infrared spectroscopy investigation of depression in patients. BMC Psychiatry. (2023) 23:217. doi: 10.1186/s12888-023-04694-z 36997897 PMC10064712

[34] Yeung MK Lin J. Probing depression, schizophrenia, and other psychiatric disorders using fNIRS and the verbal fluency test: A systematic review and meta-analysis. J Psychiatr Res. (2021) 140:416–35. doi: 10.1016/j.jpsychires.2021.06.015 34146793

[35] He J Yan S Song Z Lu Q Zhong S Lai S. Similarities and differences in working memory and neurometabolism of obsessive-compulsive disorder and major depressive disorder. J Affect Disord. (2022) 311:556–64. doi: 10.1016/j.jad.2022.05.069 35588910

[36] Shan Y Jia Y Zhong S Li X Zhao H Chen J. Correlations between working memory impairment and neurometabolites of prefrontal cortex and lenticular nucleus in patients with major depressive disorder. J Affect Disord. (2018) 227:236–42. doi: 10.1016/j.jad.2017.10.030 29102838

[37] American Psychiatric Association. Diagnostic and Statistical Manual of Mental Disorders. 5th ed. Washington, DC: American Psychiatric Association (2013).

[38] Beck AT Steer RA Brown G. Manual for the Revised Beck Depression Inventory. San Antonio, TX: Psychological Corporation (1996).

[39] Hamilton A. A rating scale for depression. J Neurol Neurosurg Psychiatry (1960) 23:56–62. 14399272 10.1136/jnnp.23.1.56 PMC495331

[40] Yang X Ma X Huang B Sun G Zhao L Lin D. Gray matter volume abnormalities were associated with sustained attention in unmedicated major depression. Compr Psychiatry. (2015) 63:71–9. doi: 10.1016/j.comppsych.2015.09.003 26555494

[41] Yang X Kumar P Nickerson LD Du Y Wang M Chen Y. Identifying subgroups of major depressive disorder using brain structural covariance networks and mapping of associated clinical and cognitive variables. Biol Psychiatry Glob Open Sci. (2021) 1:135–45. doi: 10.1016/j.bpsgos.2021.04.006 PMC9616319 36324992

[42] Rizvi SJ Quilty LC Sproule BA Cyriac A Michael Bagby R Kennedy SH. Development and validation of the Dimensional Anhedonia Rating Scale (DARS) in a community sample and individuals with major depression. Psychiatry Res. (2015) 229:109–19. doi: 10.1016/j.psychres.2015.07.062 26250147

[43] Lin J Su Y Rizvi SJ Jagoda J Li J Wu Y. Define and characterize the anhedonia in major depressive disorder: An explorative study. J Affect Disord. (2022) 313:235–42. doi: 10.1016/j.jad.2022.06.082 35788366

[44] Kaser M Foley ÉM Khandaker GM. Neurocognitive Performance in Depressed Patients with low-grade inflammation and somatic symptoms. Brain Behav Immun Health. (2022) 19:100409. doi: 10.1016/j.bbih.2021.100409 35036964 PMC8749189

[45] Santosa H Zhai X Fishburn F Sparto PJ Huppert TJ. Quantitative comparison of correction techniques for removing systemic physiological signal in functional near-infrared spectroscopy studies. Neurophotonics. (2020) 7:035009. doi: 10.1117/1.NPh.7.3.035009 32995361 PMC7511246

[46] Semkovska M Quinlivan L O’Grady T Johnson R Collins A O’Connor J. Cognitive function following a major depressive episode: a systematic review and meta-analysis. Lancet Psychiatry. (2019) 6:851–61. doi: 10.1016/S2215-0366(19)30291-3 31422920

[47] Baune BT Miller R McAfoose J Johnson M Quirk F Mitchell D. The role of cognitive impairment in general functioning in major depression. Psychiatry Res. (2010) 176:183–9. doi: 10.1016/j.psychres.2008.12.001 20138370

[48] Birnbaum HG Kessler RC Kelley D Ben-Hamadi R Joish VN Greenberg PE. Employer burden of mild, moderate, and severe major depressive disorder: mental health services utilization and costs, and work performance. Depression Anxiety. (2010) 27:78–89. doi: 10.1002/da.v27:1 19569060

[49] McIntyre RS Soczynska JZ Woldeyohannes HO Alsuwaidan MT Cha DS Carvalho AF. The impact of cognitive impairment on perceived workforce performance: results from the International Mood Disorders Collaborative Project. Compr Psychiatry. (2015) 56:279–82. doi: 10.1016/j.comppsych.2014.08.051 25439523

[50] Manohar SG Husain M. Human ventromedial prefrontal lesions alter incentivisation by reward. Cortex. (2016) 76:104–20. doi: 10.1016/j.cortex.2016.01.005 PMC4786053 26874940

[51] Nusslock R Alloy LB. Reward processing and mood-related symptoms: An RDoC and translational neuroscience perspective. J Affect Disord. (2017) 216:3–16. doi: 10.1016/j.jad.2017.02.001 28237133 PMC6661152

[52] Pizzagalli DA Iosifescu D Hallett LA Ratner KG Fava M. Reduced hedonic capacity in major depressive disorder: evidence from a probabilistic reward task. J Psychiatr Res. (2008) 43:76–87. doi: 10.1016/j.jpsychires.2008.03.001 18433774 PMC2637997

[53] Reilly EE Whitton AE Pizzagalli DA Rutherford AV Stein MB Paulus MP. Diagnostic and dimensional evaluation of implicit reward learning in social anxiety disorder and major depression. Depress Anxiety. (2020) 37:1221–30. doi: 10.1002/da.23081 32906219

[54] Rolls ET Kringelbach ML de Araujo IE. Different representations of pleasant and unpleasant odours in the human brain. Eur J Neurosci. (2003) 18:695–703. doi: 10.1046/j.1460-9568.2003.02779.x 12911766

[55] Keedwell PA Andrew C Williams SC Brammer MJ Phillips ML. The neural correlates of anhedonia in major depressive disorder. Biol Psychiatry. (2005) 58:843–53. doi: 10.1016/j.biopsych.2005.05.019 16043128

[56] Pizzagalli DA Holmes AJ Dillon DG Goetz EL Birk JL Bogdan R. Reduced caudate and nucleus accumbens response to rewards in unmedicated individuals with major depressive disorder. Am J Psychiatry. (2009) 166:702–10. doi: 10.1176/appi.ajp.2008.08081201 PMC2735451 19411368

[57] Zhang B Rolls ET Wang X Xie C Cheng W Feng J. Roles of the medial and lateral orbitofrontal cortex in major depression and its treatment. Mol Psychiatry. (2024) 29:914–28. doi: 10.1038/s41380-023-02380-w 38212376

[58] Rolls ET. The neuroscience of emotional disorders. Handb Clin Neurol. (2021) 183:1–26. doi: 10.1016/B978-0-12-822290-4.00002-5 34389113

[59] Seo H Lee D. Temporal filtering of reward signals in the dorsal anterior cingulate cortex during a mixed-strategy game. J Neurosci. (2007) 27:8366–77. doi: 10.1523/JNEUROSCI.2369-07.2007 PMC2413179 17670983

[60] Kennerley SW Walton ME Behrens TE Buckley MJ Rushworth MF. Optimal decision making and the anterior cingulate cortex. Nat Neurosci. (2006) 9:940–7. doi: 10.1038/nn1724 16783368

[61] Wallis JD Miller EK. Neuronal activity in primate dorsolateral and orbital prefrontal cortex during performance of a reward preference task. Eur J Neurosci. (2003) 18:2069–81. doi: 10.1046/j.1460-9568.2003.02922.x 14622240

[62] Rudebeck PH Walton ME Smyth AN Bannerman DM Rushworth MF. Separate neural pathways process different decision costs. Nat Neurosci. (2006) 9:1161–8. doi: 10.1038/nn1756 16921368

[63] Rushworth MF Noonan MP Boorman ED Walton ME Behrens TE. Frontal cortex and reward-guided learning and decision-making. Neuron. (2011) 70:1054–69. doi: 10.1016/j.neuron.2011.05.014 21689594

[64] Michelle C Tommy N Lauren A David S. T109. Reward-dependent connectivity with orbitofrontal cortex in subclinical depression. Biol Psychiatry. (2018) 83:S170–1. doi: 10.1016/j.biopsych.2018.02.445

[65] Aizenstein HJ Butters MA Figurski JL Stenger VA Reynolds CF 3rd Carter CS. Prefrontal and striatal activation during sequence learning in geriatric depression. Biol Psychiatry. (2005) 58:290–6. doi: 10.1016/j.biopsych.2005.04.023 16018981

[66] Kennerley SW Wallis JD. Reward-dependent modulation of working memory in lateral prefrontal cortex. J Neurosci. (2009) 29:3259–70. doi: 10.1523/JNEUROSCI.5353-08.2009 PMC2685205 19279263

[67] Aron AR Robbins TW Poldrack RA. Inhibition and the right inferior frontal cortex: one decade on. Trends Cognit Sci. (2014) 18:177–85. doi: 10.1016/j.tics.2013.12.003 24440116

[68] Hampshire A Chamberlain SR Monti MM Duncan J Owen AM. The role of the right inferior frontal gyrus: inhibition and attentional control. Neuroimage. (2010) 50:1313–9. doi: 10.1016/j.neuroimage.2009.12.109 PMC2845804 20056157

[69] Briceño EM Weisenbach SL Rapport LJ Hazlett KE Bieliauskas LA Haase BD. Shifted inferior frontal laterality in women with major depressive disorder is related to emotion-processing deficits. Psychol Med. (2013) 43:1433–45. doi: 10.1017/S0033291712002176 PMC4380502 23298715

[70] Jenkins LM Kassel MT Gabriel LB Gowins JR Hymen EA Vergés A. Amygdala and dorsomedial hyperactivity to emotional faces in youth with remitted Major Depression. Soc Cognit Affect Neurosci. (2016) 11:736–45. doi: 10.1093/scan/nsv152 PMC4847691 26714574

[71] Treadway MT Zald DH. Reconsidering anhedonia in depression: lessons from translational neuroscience. Neurosci Biobehav Rev. (2011) 35:537–55. doi: 10.1016/j.neubiorev.2010.06.006 PMC3005986 20603146

[72] Bylsma LM Morris BH Rottenberg J. A meta-analysis of emotional reactivity in major depressive disorder. Clin Psychol Rev. (2008) 28:676–91. doi: 10.1016/j.cpr.2007.10.001 18006196

[73] Kaser M Zaman R Sahakian BJ. Cognition as a treatment target in depression. Psychol Med. (2017) 47:987–9. doi: 10.1017/S0033291716003123 27938430

[74] Treadway MT Bossaller NA Shelton RC Zald DH. Effort-based decision-making in major depressive disorder: a translational model of motivational anhedonia. J Abnorm Psychol. (2012) 121:553–8. doi: 10.1037/a0028813 PMC3730492 22775583

[75] Zou YM Ni K Wang YY Yu EQ Lui SSY Zhou FC. Effort-cost computation in a transdiagnostic psychiatric sample: Differences among patients with schizophrenia, bipolar disorder, and major depressive disorder. Psych J. (2020) 9:210–22. doi: 10.1002/pchj.316 31692266

[76] Subramaniapillai M Mansur RB Zuckerman H Park C Lee Y Iacobucci M. Association between cognitive function and performance on effort based decision making in patients with major depressive disorder treated with Vortioxetine. Compr Psychiatry. (2019) 94:152113. doi: 10.1016/j.comppsych.2019.07.006 31404802

[77] Zhu Y Quan W Wang H Ma Y Yan J Zhang H. Prefrontal activation during a working memory task differs between patients with unipolar and bipolar depression: A preliminary exploratory study. J Affect Disord. (2018) 225:64–70. doi: 10.1016/j.jad.2017.07.031 28797920

[78] Steinmann S Tiedemann KJ Kellner S Wellen CM Haaf M Mulert C. Reduced frontocingulate theta connectivity during emotion regulation in major depressive disorder. J Psychiatr Res. (2024) 173:245–53. doi: 10.1016/j.jpsychires.2024.03.022 38554620

[79] Lang X Wen D Li Q Yin Q Wang M Xu Y. fNIRS evaluation of frontal and temporal cortex activation by verbal fluency task and high-level cognition task for detecting anxiety and depression. Front Psychiatry. (2021) 12:690121. doi: 10.3389/fpsyt.2021.690121 34267690 PMC8277106

[80] Ochsner KN Ray RD Cooper JC Robertson ER Chopra S Gabrieli JD. For better or for worse: neural systems supporting the cognitive down- and up-regulation of negative emotion. Neuroimage. (2004) 23:483–99. doi: 10.1016/j.neuroimage.2004.06.030 15488398

[81] Ochsner KN Gross JJ. The cognitive control of emotion. Trends Cognit Sci. (2005) 9:242–9. doi: 10.1016/j.tics.2005.03.010 15866151

[82] Beck AT. The evolution of the cognitive model of depression and its neurobiological correlates. Am J Psychiatry. (2008) 165:969–77. doi: 10.1176/appi.ajp.2008.08050721 18628348

[83] Thérond A Pezzoli P Abbas M Howard A Bowie CR Guimond S. The efficacy of cognitive remediation in depression: A systematic literature review and meta-analysis. J Affect Disord. (2021) 284:238–46. doi: 10.1016/j.jad.2021.02.009 33631438

[84] Gard DE Gard MG Kring AM John OP. Anticipatory and consummatory components of the experience of pleasure: A scale development study. J Res Pers. (2006) 40(6):1086–102. doi: 10.1016/j.jrp.2005.11.001

