# Supplementary material for: Relationship of prefrontal cortex activity with anhedonia and cognitive function in major depressive disorder: an fNIRS study
Source: Front Psychiatry. 2024 Sep 19;15:1428425. doi: 10.3389/fpsyt.2024.1428425 (PMC11450226; doi:10.3389/fpsyt.2024.1428425)
Supplement: Supplementary file 1 [file DataSheet1.pdf]

Supplementary Table 1.

Correlation between cognitive function and prefrontal activity in the MDD group.

items	CH4
SWM_BE	*r* = -0.07, *p* = 0.66
SWM_TE	*r* = -0.06, *p* = 0.66
SWM_str	*r* = -0.06, *p* = 0.66
RVP_A	***r* = 0.31, *p* = 0.03**
RVP_TH	***r* = 0.34, *p* = 0.03**
RVP_TM	***r* = -0.33, *p* = 0.03**
RVP_PH	***r* = 0.34, *p* = 0.03**

Note: *p* value was corrected by FDR correction

Supplementary Table 2.

Correlation between cognitive function and anhedonia in the MDD group.

items	DARS_motivation	DARS_effort
SWM_BE	*r* = -0.27, *p* = 0.06	*r* = -0.30, *p* = 0.09
SWM_TE	*r* = -0.26, *p* = 0.06	*r* = -0.29, *p* = 0.09
SWM_str	*r* = -0.21, *p* = 0.12	*r* = -0.18, *p* = 0.31
RVP_ A’	***r* = 0.34, *p* = 0.03**	*r* = 0.19, *p* = 0.31
RVP_TH	***r* = 0.32, *p* = 0.03**	*r* = 0.13, *p* = 0.31
RVP_TM	***r* = -0.33, *p* = 0.03**	*r* = -0.14, *p* = 0.31
RVP_PH	***r* = 0.32, *p* = 0.03**	*r* = 0.14, *p* = 0.31

Note: *p* value was corrected by FDR correction

Supplementary Table 3.

Correlation between anhedonia and prefrontal activity in the MDD group.

items	CH4
DARS	***r* = 0.30, *p* = 0.04**
DARS_consume	*r* = 0.28, *p* = 0.05
DARS_effort	*r* = 0.24, *p* = 0.08
DARS_desire	***r* = 0.30, *p* = 0.04**
DARS_motivation	***r* = 0.31, *p* = 0.04**

Note: *p* value was corrected by FDR correction

List of abbreviations

DARS, Dimensional Anhedonia Rating Scale; DARS_consume, hedonic consume score of DARS; DARS_effort, hedonic effort score of DARS; DARS_desire, hedonic desire score of DARS; DARS_motivation, hedonic motivation score of DARS
RVP, Rapid Visual Information Processing; A’, is the signal detection measure of sensitivity to the target, regardless of response tendency (range 0.00 to 1.00; bad to good); TH, total hits; TM, total mises; PH, probability of hit; SWM, spatial working

memory; BE, times the subject revisits a box in which a token has previously been found; TE, This is the number of times a box is selected that is certain not to contain a blue token and therefore should not have been visited by the subject; strategy: higher scores indicating inferior neurocognitive performance.
